# Remarks on Abdominal Ptosis, Viscero-Ptosis, Entero-Ptosis, or Glenard’s Disease*Read before Richmond Academy of Medicine and Surgery, March 24, 1898.

**Published:** 1896-05

**Authors:** Hugh M. Taylor

**Affiliations:** Richmond, Va.


### REMARKS ON ABDOMINAL PTOSIS, VISCERO-PTOSIS, ENTERO-PTOSIS, OR GLENARD'S DISEASE.*

By HUGH M. TAYLOR, M.D.,

Richmond, Va.

The occurrence, very recently in my practice, of two cases of displaced kidney with well marked renal or Dietl's crises, awakened an interest in that curious and little understood condition—abdominal ptosis or displacement or prolapse of some one or all of the abdominal viscera. A due appreciation of the clinical importance of viscero-ptosis has been increasing since Glenard's study of the subject.

Recently Mr. Treves has brought it prominently to the attention of the profession, and considered at length the surgical aspect of its treatment.

The cause (or causes) of viscero-ptosis is somewhat a mystery. It is met with more frequently in women, notably women who have borne children. Pregnancy, therefore, seems to occupy an important etiological relation. Prolonged vomiting from any cause may be a causative factor, as may also injuries, prolonged coughing, straining, lowered vitality, general relaxation and absorption of fat. However induced, abdominal or visceral-ptosis is attended by relaxation of the abdominal muscles and elongation of the ligaments supporting or holding in position the abdominal viscera. This stretching of the ligaments and prolapse of the viscera may be limited in extent or occur to such a degree as to give rise to that common condition, movable, floating, or displaced kidney, or the less common condition of floating liver, or the perhaps more common, but less frequently recognized, displacement of the hepatic flexure of the colon and with it the pyloric end of the stomach, liver, etc.

This anatomic pathological change, culminating in viscero-ptosis, usually begins in the right colic angle, and is attended by a gradual descent of the hepatic flexure of the colon until it finally occupies

*Read before Richmond Academy of Medicine and Surgery, March 24, 1896.

a lower plane in the abdominal cavity. With this descent of the colon, the pylorus is displaced and flexed, its lumen contracted, the contents of the stomach are prevented from passing readily into the duodenum, accumulation and decomposition of the stomach contents results, and entails as a further consequence pain, nausea, vomiting, fermentation, dilatation of the stomach, and a long-continued train of gastric disturbances which are unsuccessfully treated by many physicians in many ways before the true causative agent is recognized. Flexion and obstruction to the passage of the duodenal contents to the jejunum, a fecal stasis with its many injurious consequences may result from prolapse of the right-angle of the colon and traction on the duodenum, and to the stomachic we may have intestinal disorders added.

A displaced liver and bile tract may interfere with the latter's function as a drain tract. Forty ounces of bile are discharged per diem; even a partial obstruction by flexion of this tract may favor inspissation, cholelithiasis, cholangitis, or cholecystitis, and to a greater or less extent, possibly cholemia.

Many patients with viscero-ptosis suffer no inconvenience whatever. A majority of those so affected, if they are troubled at all, are so little affected that they are not deprived of comparative good health and comfort. But a good many more than we have in the past so credited, are rendered invalids in this way. In the main such patient's standard of health is below par; they lose their appetite, flesh, and strength, become neurasthenics, experience a sense of weight or dragging in the back and abdomen. When in the upright posture they suffer pain, nausea, vomiting, and numerous vague nervous manifestations, and there may be alternate attacks of diarrhea or constipation, the latter amounting in exceptional instances almost to obstruction.

This condition may induce neurasthenia, or on the other hand, if the ptosis abdominalis occurs in a neurasthenic patient, the discomforts incident to the ptosis are very much intensified.

In not a few instances the condition of chronic invalidism is so irremediable that operative interference is the only curative resource we can offer.

Celiotomy, and shortening by suturing the elongated ligaments, has furnished a sufficient number of successes to stamp it a warranted surgical procedure.

Exaggerated viscero-ptosis is typified in floating, displaced, or movable kidney or liver; the former (floating kidney) a common occurrence; the latter (prolapsed liver) very much less so and less serious. Few practitioners of experience who will look for them, will fail to find quite a number of cases of displaced kidney; happily a very large majority of such cases are neither discomfort nor disease-producers.

The experience of most observers accords with that of Mr. Treves and sanctions the classification of displaced kidneys clinically under three heads.

I. Patients who have displaced kidneys, do not know it, and suffer no inconvenience whatever from it. This type represents by far the largest class.

II. Patients with displaced kidneys, who may or may not know of it, who suffer from gastro-intestinal discomfort and perhaps more frequently a long train of vague neurotic disturbances.

III. Patients with movable kidney, who are the subjects of occasional or frequent mild or severe attacks of Dietl's or renal crises.

The first, the largest class, are never, the second may be, the third, with few exceptions, must be legitimate subjects for operative measures or nephrorrhaphy. In some instances the movable kidney is attended all of the time with gastro-enteric and neurotic manifestations, and only occasionally or from time to time with the condition of Dietl's or renal crises. This fact is well illustrated by a case now under his observation.

An unmarried woman from Eastern North Carolina has experienced for several years, several times a month, sometimes not for several months, violent paroxysmal attacks of pain focused in the right hypochondriac or lumbar region and attended by persistent vomiting and subsequent local soreness for several days. For years she has been treated for gall-stones and hepatic colic. Her appetite, digestion, and nervous system ha vesuffered seriously, and obstinate constipation has been the rule. She has gradually lapsed into a condition of semi-invalidism and neurasthenia. With the train of symptoms presented by this case in its beginning, few practitioners would have failed to associate the manifestations with some morbid condition of the gall-tract, most probably cholelithiasis, but at the present time the mobile kidney can be displaced from its nest and made to prolapse almost into the pelvis; and through the thin abdominal walls its shape can be plainly outlined. The attacks are clearly typical Dietl's or renal crises. This assertion is made with a due appreciation of the fact that we are not warranted in every instance in pronouncing a tumor associated with the above mentioned symptoms unmistakably a displaced kidney. Able clinicians record frequent diagnostic mistakes in this particular field.

The renal crises simulate closely nephritic colic from stone, hepatic colic, or even acute intestinal obstruction, and from all of these a differential diagnosis must be made.

Nephrorrhaphy is clearly indicated in this case and will be performed first, because of the discomfort incident to the frequent renal crises, and second because of the long-continued gastro-enteric and neurasthenic disorders.

There is a diversity of opinion as to what produces the renal crises. In the opinion of some, it is due to torsion or twisting of the pedicle and disturbed vascular and nerve supply. Another, and perhaps correct, assumption is, that it is due to flexion and twisting of the ureter and damming back of the urine, with over-distention of the pelvis and calices of the kidney.

In the second case under observation the violent attacks of pain and vomiting were at first attributed by himself to gall stones in the gall-bladder or cystic duct, as there was no jaundice. The kidney in the early history of the case, before it had wandered a great distance from under the surface of the liver, was mistaken for a distended gall-bladder; there is now such a degree of prolapse that all obscurity in the diagnosis has been removed. No operative procedure has been contemplated in this case because of the extreme age, enfeebled condition of the patient, and because of a serious, long-continued chronic bronchitis. A year or more ago this patient had frequent attacks, but had escaped for many months until within the past week or ten days. The recent attack ensued almost immediately after a very gentle manual examination of the displaced viscus and was obviously due to a twist of the pedicle brought on by the examination. The pain, vomiting, etc., incident to the attack were sufficiently severe to call for repeated doses of morphia for its relief, and as usual in such cases there was persistent soreness for five or six days. Nephrorrhaphy is only contraindicated in this case by the age and feeble condition of the patient and the fact that the crises have been infrequent for the past six or eight months.

To ascertain the existence of ptosis of any of the abdominal viscera, an examination should be made with the patient in the erect or semi-erect position. On assuming the recumbent position, the organs gravitate back to their normal position. In extreme ptosis of the kidney, the viscus can be made to prolapse by instructing the patient to cough, or by pressure of the examiner's hands.

To a very marked extent the discomforts incident to such a condition are relieved when the patient is lying down, and to a limited extent they are ameliorated by mechanical support in the shape of abdominal binders.

When the patient is lying down nothing unusual is noticed in the appearance of the abdomen, but when the patient with general ptosis assumes the upright posture, the upper portion of the abdominal cavity is preternaturally flat, the pelvis unduly prominent.

A better understanding of the clinical significance of abdominal ptosis, including that of the kidney, would widen the field of legitimate abdominal surgery and limit to some extent the scope of dietetic and gastro-intestinal therapy, and indicate the origin of not a few obscure neurotic disturbances.

---

AN author should say all he has to say in the fewest possible words, or his reader is sure to skip them; and in the plainest possible words, or his reader will certainly misunderstand them. Generally, also, a downright fact may be told in a plain way; and we want downright facts at present more than anything else.—*Ruskin.*

